# Experiences of At-Risk Women in Accessing Breastfeeding Social Support During the Covid-19 Pandemic

**DOI:** 10.1177/08903344221091808

**Published:** 2022-04-25

**Authors:** Emila Siwik, Samantha Larose, Dalia Peres, Kimberley T. Jackson, Shauna M. Burke, Tara Mantler

**Affiliations:** 1 Health and Rehabilitation Sciences Program, Faculty of Health Sciences, The University of Western Ontario, London, ON, Canada; 2 Arthur Labatt Family School of Nursing, Faculty of Health Sciences, The University of Western Ontario, London, ON, Canada; 3 Health Sciences with Biology Program, Faculty of Health Sciences, The University of Western Ontario, London, ON, Canada; 4 School of Health Studies, Faculty of Health Sciences, The University of Western Ontario, London, ON, Canada

**Keywords:** Abuse Assessment Screen, at-risk mothers, breastfeeding, Canada, COVID-19, intimate partner violence, mixed methods research, postpartum, social support

## Abstract

**Background:**

With strict public health measures implemented in March 2020 due to the COVID-19 pandemic, many breastfeeding parents, who are within an at-risk population, have experienced limited formal and/or informal breastfeeding social support. In the Canadian context, the experiences of these women is unknown.

**Research Aim:**

To explore the experiences of at-risk postpartum breastfeeding women in accessing formal and informal breastfeeding social support during the COVID-19 pandemic.

**Methods:**

This was a prospective, longitudinal interpretive description study using mixed methods. Data were gathered using an online survey and one 52–112-min semi-structured interview at 12-weeks postpartum. At-risk breastfeeding participants were those who lack social support and had at least one of the following: age < 25 years; experiencing or had experienced intimate partner violence; or of low income. We sought participants’ experiences of accessing breastfeeding social support during the first few months of the COVID-19 pandemic/lockdown. Seven participants completed the survey and the interview.

**Results:**

Participants identified that the COVID-19 pandemic created barriers to accessing formal and informal breastfeeding social support, which stemmed from public health restrictions and difficulties communicating online with families and healthcare providers. Additionally, participants identified that the COVID-19 pandemic/lockdowns facilitated feelings of connectedness, protection, and resiliency.

**Conclusion:**

We provide preliminary insight into the experiences of trying to access breastfeeding social support during the COVID-19 pandemic. Future researchers should seek to prioritize improved communication and resources in supporting breastfeeding during COVID-19 and future pandemics/lockdowns.

**Key Messages**

- Despite preliminary evidence, the influence of the COVID-19 pandemic on breastfeeding social support among those who are at-risk in Canada remains unknown.
- Participants expressed barriers to breastfeeding, including a lack of in-person contact and challenges of communicating online.
- Participants reported that breastfeeding social support facilitated their connectedness, protectiveness, and resiliency in facing challenges.

## Background

Breastfeeding is considered the gold standard for optimal infant feeding (World Health Organization [WHO], 2020). The WHO (2020) and Health Canada (Public Health Agency of Canada, 2018) have recommended infants be exclusively breastfed for the first 6 months of life and that breastfeeding be continued until 2 years of age and beyond. Currently, only 30% of Canadian mothers exclusively breastfeed their infants, and of those 47% cease exclusive breastfeeding before their infant is 6 months of age (Francis et al., 2020). Within Ontario, only 53.2% of mothers exclusively breastfeed their infant to 6 months of age (Baby-Friendly Initiative Ontario, 2019).

Women from “at-risk populations” are more likely not to breastfeed or to cease breastfeeding prematurely (Dennis, 2002). The criteria for at-risk population were identified by Dennis (2002) who highlighted that women who were young, lacked breastfeeding social support, and were of low socioeconomic status were at a heightened risk of prematurely ceasing breastfeeding practices. In addition, violence was included in the criteria as 25%–30% of Canadian women experience intimate partner violence (IPV) at some point in their lifetime (Burnett et al., 2016). Thus, exploring and identifying challenges, as well as strategies that promote breastfeeding for at-risk women is an important public health priority.

Access to formal and informal breastfeeding social support is essential to breastfeeding initiation and duration for at-risk women (Brockway et al., 2017). Formal breastfeeding social support, including healthcare and informational resources provided to women by healthcare providers and breastfeeding professionals, has been identified as highly effective in promoting the intention to breastfeed in at-risk women (Dennis, 2002; McFadden et al., 2017). Women who receive breastfeeding social support from healthcare professionals are more likely to initiate breastfeeding and to continue breastfeeding longer than women who do not receive formal support (Lee et al., 2019; Miller-Graff et al., 2018). Informal breastfeeding social support from family and friends also has been shown to contribute to breastfeeding success. For example, Bano-Pinero and colleagues (2018) found that participants who communicated their breastfeeding challenges and doubts within breastfeeding social support networks were more likely to breastfed longer, compared to participants who did not have informal social support available.

The COVID-19 pandemic and resultant public health guidelines of physical distancing has implications for infants and mothers (Loewenthal et al., 2020). In a recent study by Zanardo and colleagues (2021), they reported that the COVID-19 pandemic and associated lockdown in northeastern Italy contributed to a reduction in exclusive breastfeeding by 15% compared to women who gave birth in the previous year. Moreover, the COVID-19 pandemic initially shuttered many formalized breastfeeding social support services such as clinic appointments and breastfeeding social support groups (Connor et al., 2020; Gribble et al., 2020; Pérez-Escamilla et al., 2020). Moreover, throughout the COVID-19 pandemic, access to family and friends for breastfeeding social support has been limited, as stay-at-home orders and visiting restrictions have prevented women from getting together with support networks (Spatz & Froh, 2021). Unfortunately, restricted access to informal supports can result in women experiencing severe isolation, depression, and anxiety during the postpartum period (Silverman et al., 2020; Viswanath & Mullins, 2020). Further, the breastfeeding guidance, support, and reassurance that women typically receive from their family and friends have been greatly decreased during the pandemic, potentially resulting in decreased breastfeeding self-efficacy and poorer breastfeeding outcomes (Alemeida et al., 2020).

Together, the loss of formal and informal breastfeeding social support could have a detrimental influence on breastfeeding, particularly among at-risk women; however, there are limited data available to explore these influences on breastfeeding social support, as most researchers have been focusing on how services were reduced and the resultant emotional toll on mothers. A study by Viswanath and Mullins (2020) that reported participants’ access to maternal and postnatal health services was limited, as many maternal and infant clinics were converted for COVID-19 testing and inpatient care. This lack of formal breastfeeding social support led many participants to experience elevated levels of frustration and stress, creating a perceived reduction in milk supply and breastfeeding confidence (Viswanath & Mullins, 2020). Additionally, Synder and Worlton (2021) noted that mothers had limited access to lactation support and informal care groups during the COVID-19 pandemic and that mothers emphasized the desire for more in-person formal and informal support around the areas of practical aid (e.g., how to hold the infant to ensure a proper latch). Spatz and Froh (2021) further observed that limited support resulted in participants experiencing fear associated with breastfeeding their infant, ensuring their infant was healthy, and the constant and ever-changing nature of the pandemic and its influence on the community. As a result, these participants were more likely to experience hardships related to initiating or continuing their breastfeeding practices (Spatz & Froh, 2021). Based on the limited literature related to breastfeeding practices and access to care during the COVID-19 pandemic for at-risk women, it is imperative to explore the breastfeeding care that did continue during the COVID-19 pandemic/lockdown.

The aim of this study was to explore the experiences of at-risk postpartum breastfeeding women in accessing formal and informal breastfeeding social support during the COVID-19 pandemic. To our knowledge, this study is the first of its kind to provide an in-depth exploration of at-risk women in accessing breastfeeding social support during the COVID-19 pandemic, acknowledging the importance this phenomenon has on breastfeeding experiences and outcomes.

## Methods

### Research Design

This was a prospective, longitudinal interpretive description study using mixed methods. An interpretive description (ID) was selected as it affords the exploration of socially and culturally constructed phenomenon (Thorne, 2008). ID encourages researchers to bring meaning to the interaction between participants and phenomena and embeds study findings in the context of the needed changes to practice (Thorne, 2008). Participants were recruited from the broader project (*Engaging Mothers in a Breastfeeding Intervention to Promote Relational-Attachment, Child Health, and Empowerment* [EMBRACE]) conducted in Ontario, Canada. This study was approved by an Institutional Review Board at an urban university in Southwestern, Ontario on May 29, 2019 (#113464).

### Setting and Relevant Context

This study took place at a physician-led urban postpartum clinic in Southwestern, Ontario. This clinic specializes in infant and maternal health, breastfeeding and postpartum care within the 1st year of an infant’s life. This Southwestern city has approximately half a million residents, one birthing hospital, and over 140,000 yearly provide-wide births. In 2012–2018, breastfeeding initiation in the province of Ontario increased by 6% from 77.7% to 84.2%, while exclusive breastfeeding rates increased by 1.5% from 51.9% to 53.2% (Baby-Friendly Initiative Ontario, 2019).

### Sample

The target population included participants from an at-risk population and attending the physician-led urban postpartum clinic in Southwestern, Ontario. All participants identified as female. Eligible participants were: (1) at least 18 years of age; (2) able to speak and read in English; (3) receiving breastfeeding care from the clinic during the March to September 2020 COVID-19 lockdowns; and (4) from an at-risk population. An additional criterion of access to a telephone and the internet was added so data could be collected remotely. Exclusion criteria included: (1) any participants with physical health challenges that interfered with their ability to breastfeed (e.g., participants who experienced nipple issues); and (2) participants who joined the urban postpartum clinic after 12 weeks postpartum, as the data were collected at 12 weeks postpartum.

The sample included a total of seven participants (*N* = 7), as two were lost to follow-up and did not complete the interview. This sample was selected as it is sufficient to answer the research question. The sample size is consistent with similar studies using ID including Spurr and colleagues (2021). According to Thorne (2008), a small sample size anywhere from five to 12 participants is sufficient to gather meaningful results.

### Measurement

The on-line survey was used to collect demographic data, and the semi-structured interviews explored participants’ experiences of accessing breastfeeding social support during the first few months of the COVID-19 pandemic/lockdown.

#### Demographics

Participant demographics (e.g., age, ethnicity, marital status, education, employment, and income) and infant demographics (e.g., age, weight, sex, delivery methods, and if the infant was breastfed) were assessed at 12-weeks postpartum, as part of the online questionnaire. Infant demographic questionnaire was validated using the WHO/United Nations Children’s Fund (UNICEF) breastfeeding indicators for mothers (see supplemental material; WHO, 2009), and maternal at-risk population characteristics.

#### At Risk Population

At-risk population was operationalized as a lack of breastfeeding social support and/or at least one of the following: (1) were under the age of 25; (2) had a history of IPV; and/or (3) of low income (Dennis, 2002) outlined as having an annual family income less than $31,061 (Low-Income Cut-Off Score; Government of Canada, 2020). At-risk population characteristics were asked at 12-weeks postpartum using the online questionnaire, following participant demographics.

At risk population characteristics were assessed using the following questions with a yes/no response: Do you feel you have limited breastfeeding social support? Are you 18 years or older? Do you have a family net income of less than $31,061? Have you experienced intimate partner violence at some point in your life? For IPV, if participants answered yes then the Abuse Assessment Screen (AAS; McFarlane & Parker, 1994), a previously validated scale, was used (Cronbach’s α = 0.56). For the purposes of this study, any positive response on the AAS scale was indicative of abuse and was used to determine the number of participants who had experienced or were experiencing IPV.

#### Qualitative Interviews

Semi-structured interviews (see interview guides in the supplemental materials) had two main foci: experiences of breastfeeding social support and the influence of COVID-19. Prior to the interview start, in order to diminish social desirability bias (Larson, 2018), participants were told the following: “I want you to know that there are no right or wrong answers, we are simply interested in what is true for you.” The interviews ranged in length from 52–112 min.

### Data Collection

Recruitment and data collection occurred between March 10, 2020, and September 1, 2020. After birth (72 hr post), potential participants received a referral from the attending hospital physician, and/or contacted the clinic personally via email or telephone. Once women enrolled in the clinic, purposeful sampling was used. Clinic patients were invited to participate via questions on their intake form when they joined the care team. Interested patients were provided with a letter of information and consent, which was signed digitally. Once consent was received, participants completed a demographic and breastfeeding questionnaire immediately, and then again at 12-weeks postpartum (see supplemental material). At 12-weeks postpartum, the participants received an email from the study researchers asking if they would be interested in being interviewed. If they agreed, participants were sent the secondary demographic and breastfeeding questionnaire. Additionally, a one-on-one, telephone-based, 52–112-min semi structured interview was completed with a trained graduate. Phone interviews were recorded using a handheld device and transcribed verbatim. Honorariums ($5 CDN for completing the 12-week postpartum survey and $20 CDN for completing the interview) were provided to all participants to both recognize their contributions, and to minimize barriers to participation.

Reflexivity during the data collection and analysis was managed following the practice of self-reflection, after each interview, using journaling (Dodgson, 2019). Both the primary and secondary researchers would address their own personal thoughts, ideas, and feelings following an interview, with each being recorded in a personal journal. This encouraged the researchers to reflect on the information collected during the interview, and aided in documenting first-hand the analytical progress that follows closely with the reflexive stance adopted by the methodology of ID (Thorne, 2008).

To ensure the safety of participants, phone calls were made using an “unknown” number ensuring the call could not be traced. Additionally, the participant was able to stop the interview at any time for any reason. To ensure the confidentiality of participants, interviews were deleted immediately after transcriptions and all participants were assigned a number (e.g., 001) to maintain anonymity.

### Data Analysis

For the quantitative data, given the small sample size and lack of power, analysis consisted of measures of central tendency and dispersion. For interviews, an interpretive description (ID) approach, as described by Thone (2008), was used. Coding was done independently by three researchers. Initially, each researcher became immersed in the data and subsequently began open coding (Thorne, 2008). Next axial coding was undertaken wherein relationships between the themes that emerged from open coding were explored (Thorne, 2008; Scott & Medaugh, 2017). Finally, line by line coding was done to ensure all the data were encapsulated in the findings, paying particular attention to instances where data fell outside the emerging codes (Thorne, 2008). Once coding had been completed by each researcher, the researchers came together to discuss findings and determine if there was consensus in the emerging themes. Consensus was reached for all themes by all researchers (Table 1).

**Table 1.** Data Analysis Structure Table.

Definition	Codes	Code Definition
Barriers as a Result of the COVID-19 Pandemic: Negative impacts of the COVID-19 pandemic on formal and informal breastfeeding social support	Inability to Access Formal Breastfeeding Social Support Limited In-Person Informal Breastfeeding Support	Ways formal breastfeeding social supports were limited during the COVID-19 pandemic Ways informal breastfeeding social supports were limited during the COVID-19 pandemic
	Challenges Communicating Online	Difficulties participants faced in communicating with social supports online
Facilitators to Breastfeeding as a Result of the COVID-19 Pandemic:	Connectedness	Ways participants felt connected to their partner, friends, and healthcare teams during the COVID-19 pandemic
Positive impacts of the COVID-19 pandemic on formal and informal breastfeeding social support	Protectiveness	Ways participants limited their access to certain social supports as a result of the COVID-19 pandemic
	Resiliency	Ways participants showed strength during the COVID-19 pandemic in terms of breastfeeding

Incorporated in the collection of data and analysis were strategies to reduce researcher bias and to support data trustworthiness including note taking during the interviews, reflexive journaling throughout the data collection/analysis phases, and independent analysis of transcripts by team members (Guba & Lincoln, 1989).

## Results

### Characteristics of the Sample

Participants’ ages ranged from 27–36 years, with an average age of 29.8 years (*SD* = 3.71). Marital status was reported as single (*n* = 3; 42.9%) or married/common law/engaged (*n* = 4; 57.2%), and three participants had been with their partner for 2 or more years (*n* = 3; 42.9%). Additional participant characteristics can be found in Table 2.

**Table 2.** Demographic Information of Participants at 12-Weeks Postpartum (*N* = 7).

Characteristic	n (%)
Ethnicity	
Afghan	3 (42.9)
Jamaican	1 (14.3)
French Canadian	1 (14.3)
Indian	1 (14.3)
Multiethnic	1 (14.3)
Born in Canada	
No	5 (71.5)
Yes	2 (28.6)
Education	
Less than high school	2 (28.6)
Community college	1 (14.3)
University undergraduate	1 (14.3)
University graduate	3 (42.9)
Employment	
Employed part-time	3 (42.9)
Stay-at-home mom	2 (28.6)
Looking for paid work	1 (14.3)
Maternity leave	1 (14.3)
Income	
$20,000-$49,999	4 (57.1)
$50,000-$99,999	2 (28.6)
I prefer not to answer	1 (14.3)

All participants identified as having a lack for breastfeeding social support. No participants were under the age of 25. Three participants (42.9%) identified as having experienced and/or were experiencing IPV, per the AAS scale (Cronbach’s α = 0.51). These participants had experienced emotional and/or physical abuse by an intimate partner or someone close to them in the last 12 months. Four participants identified as being below the Low Income Cut off Score of $31,060 (57.2%; Government of Canada, 2020).

#### Infant Feeding Practices at 12 Weeks Postpartum

All seven of the participants’ infants were breastfed at the breast; however, only four were breastfed within the 1st hr after birth (57.2%). Six infants (85.8%) were still being breastfed at the 12-week postpartum questionnaire, with three infants (42.9%) being breastfed exclusively, and four infants (57.2%) not breastfed exclusively and receiving supplemental formula.

### Thematic Results

#### Theme 1: Barriers as a Result of the COVID-19 Pandemic

Theme one includes three subthemes: (a) inability to access formal breastfeeding social support; (b) limited in-person formal breastfeeding social support; and (c) challenges communicating online.

##### 1a. Inability to Access Formal Breastfeeding Social Support

Most participants said that the COVID-19 pandemic and the resulting healthcare service suspensions affected their ability to access formalized breastfeeding social support. Limited appointment times, issues accessing healthcare practitioners, and having to attend appointments alone were some of the challenges that participants expressed about their breastfeeding social support-seeking experiences. One participant (005) struggled to find a healthcare practitioner during the COVID-19 pandemic, she said:

I was looking before, as well, but they were not accepting patients and with the pandemic going on. Even the pediatricians who were accepting patients, they are. . . they put everything on hold. So even now, I am kind of in a limbo to find a good doctor for [the baby].

For some participants, the heightened restrictions of the COVID-19 pandemic also made it challenging to find healthcare providers who were willing or able to see new patients. One participant (003) described making multiple attempts to access healthcare support with limited success:

I’ve talked to so many different people because I had to call to cancel. . .. I called when they first called to get me to come [to the clinic]. I called because I was upset about a problem and, again, I have not had it dealt with. So, it’s not like I’ve been—it’s hard. Like, even when I was trying to make my 6-week appointment, calling around, and these people are just like, “No, we won’t. We can’t take you, like, sorry. It’s COVID.” And they were, like, kind of. . .. It’s hard and you don’t really know what to do, because you don’t know what’s safe.

Of those who were able to obtain and access support from healthcare practitioners, many participants experienced cancellations of their classes and appointments due to restrictions. For some, postnatal classes were cancelled, potentially resulting in gaps in knowledge that could have been beneficial to their breastfeeding experiences. One participant explained, “Some of the other [breastfeeding] classes they used to conduct regularly were cancelled. Due to the pandemic, everything was cancelled. That was one thing I missed out on” (008). This was supported by another participant (006), who said:

Because [of] all the [COVID-19] now though . . . we were not allowed to have some meetings and things like that. So, yeah, and they booked me that class with the nurse to. . . with the nurse to answer all your questions. I don’t know the name of it. Uh, but, because of [COVID-19] they had to cancel.

##### 1b. Limited In-Person Informal Breastfeeding Social Support

While the ability to access formalized breastfeeding social support from healthcare practitioners was limited, participants also struggled with access to their informal supports (e.g., partner, friends, extended family members). Access to these individuals affected participants’ experiences with breastfeeding during the pandemic. For some participants, not being able to experience in-person interactions influenced their perception of support. One participant (006) said:

Yeah, so with everything now. . . it’s just in video calls, right? I don’t know, it’s really hard to us, because of the [COVID-19], my husband has, uh, one part of his family lives in the U.S. and so, they were coming to help us in the first month, right? Uh, because [of] the [COVID-19] and all the borders were closed. And so, we didn’t have any help at all [laughter]. And, so, it was really difficult to us in the beginning because we didn’t know how to do a lot of stuff right. We are still learning, actually. We are learning, yeah.

This was also found by another participant (009) who noted that because she was able to access a trusted friend who lived in her apartment building for physical support, her experience was more positive:

Friends? Well, I do have one friend in particular that lives in my apartment building. So, yeah. I got a little support in terms of sort of having someone to talk to, or, if she comes down to the apartment I can say hold the baby for me while I do something around the house quickly.

Limited access to informal breastfeeding social support also compounded concerns about access to healthcare services. Participants, who were able to obtain and attend in-person appointments indicated that accessing care was, at times, complicated by the fact that they were expected to do this alone, without their spouse or another support person. One participant (005) described how attending her appointments without her husband was challenging due to her physical health and the logistics of visiting the clinic alone with a baby:

I mean, they’re doing their best with the [COVID-19] guidelines going on. I would have just wanted my husband with me for a couple more visits, but I do understand with the restrictions going on. I was suffering from upper back pain; I pulled a muscle. So, I was feeling that it was a bit hard to carry the car seat into the clinic and bring it back.. . . But, like, I understand with the restrictions going on.

##### 1c. Challenges Communicating Online

With access to in-person supports hindered by stay-at-home orders, participants were often limited to utilizing online forms of support. Despite the prevalence of online communication, many participants described having trouble using online support systems effectively to help with their breastfeeding. When asked if she had access to any breastfeeding social support groups, one participant (006) explained:

Yeah, the social worker helped me with that. . .. Uh, but in that time, that groups were not allowed to be together, right? Because of the COVID-19 pandemic. And, so, she sent me some online groups, but that didn’t work for me at all. So, I think, if it’s possible, when everything becomes normal again, I think I’ll try again. I want to learn from other mothers and talk with [them about] their experiences, you know? And it’s good for the baby to know other babies, I think.

For some participants, the pressures of online communication affected their perceptions of the support that they received from their friends and families. When support persons could not be present to offer in-person support, some participants felt that the online or telephone communication they received was excessive and resulted in a hindrance to their feelings of support (002):

So, my mother-in-law, she was mad because she couldn’t come to the house and help, which I understand. But uh. . . there was like constant things. They wanted to call me all through the process. They are like, “your sister-in-law has gone through this process. Do this, do this, do this.” All those things they wanted me to do without questioning.. . . And then, there was calls coming in at inappropriate times, sometimes at like midnight and very early morning. So, I was feeling like I needed a break from that.

Another participant (005) felt the pressure by not having her support persons physically present during the breastfeeding journey, she felt pressured to always be actively present online:

I don’t have a lot of family support here, personally. Everybody’s like back home, and the family that we do have here, we didn’t want anybody to come and be with us. So, everyone is like, calling and doing the video chat and asking, “How are you doing? How are you doing?” And so, like, “Are you breastfeeding? Are you breastfeeding, or not?” And so, it felt like a constant pressure on me.

When this participant was asked about what might have helped to mitigate the pressures she was experiencing, she stated, “I think that if somebody was able to be physically present, it would have been a lot more helpful.” Of the participants who were provided with online resources (e.g., external links to WHO breastfeeding practices), many acknowledged that they did not use or find them helpful. For example, one woman (008) said, “I didn’t use the, like, full content [online resources and videos], because I didn’t have time to read.”

#### Theme 2: Facilitators to Breastfeeding as a Result of the COVID-19 Pandemic

Theme two includes three subthemes: (a) connectedness; (b) protectiveness; and (c) resiliency.

##### 2a. Connectedness

The ability for a mother to feel connected to oneself, their infant, and their support systems was identified as important and a priority for many of the participants interviewed. Specifically, the COVID-19 pandemic allowed participants in this study to spend important quality time with their infants, increasing their perceived connectedness. One participant (007) was asked what made her postpartum experience easier, she replied that the pandemic allowed her to slow down and shift her focus onto her baby:

Yeah, and especially again during the pandemic, right? Your mind is also on your baby but also on the world around you, and you’re trying to do your best. To be honest, the pandemic hit and having the shutdown because you’re just at home, focusing on growing the baby.

Echoing this, another participant (006) found that the quarantine periods allowed her to feel more connected with her partner as they navigated the experiences of becoming new parents together:

Actually, I was grateful about the quarantine and, uh, about the borders [being] closed, because it was really hard for us to be alone, too, but it was great because we figured out how to work together, uh, my husband and I, you know? And, so, I think this was really great because my husband is really present right now for the baby.

Connectedness was noted between participants and their healthcare teams as well. One participant (003) spoke about how calls from her healthcare team made her feel like a priority and increased her feelings of connectedness with them: “Just being able to have people who you don’t know call, and who genuinely care. And who are advocating for you to come in, like, for your mental health, and that’s beautiful.”

##### 2b. Protectiveness

With the threat of virus transmission and fear of becoming infected, many participants described feeling protective of themselves, their infant, and their families during the pandemic. In many instances, this resulted in participants limiting their own access to certain supports they might have otherwise actively sought out and/or utilized, in order to reduce the risk of becoming exposed to the virus. When asked about her access to breastfeeding social support, one participant (006) explained:

Yeah, our family, we, we—actually, we’re used to talking with them with video calls because of all the [COVID-19] situation, right? And because they’re. . .far away from us, too. And, uh, we communicate. I didn’t try to find any [formalized] support yet, because I was not feeling safe to bring him to any group or anything. Actually, I’m not, I’m still not feeling safe to bring him outside yet.

While fears of the COVID-19 virus hindered access in some cases, one participant (003) described that her healthcare provider’s concern for her wellbeing aided in reducing her anxieties during the COVID-19 pandemic: “They were very sweet. They were asking about my wellbeing, and they said that I mattered, and it was right when COVID came out.” Further, she described how protective protocols and an attentiveness to her, and her infant resulted in feelings of safety and comfort when accessing in-person care:

They were like, “No problem, come in here,” and “we’ll call you.” Like, it’s very—They were so careful with during COVID-19 pandemic, and I was the only one in there. I came in and, again, the nurses and staff were so fantastic. They handled my anxiety for sure. I am very grateful that they took me in, and it was very safe.

When support systems adhered to safety protocols, participants described feeling more comfortable and less anxious when accessing them. When asked if the COVID-19 pandemic protocols negatively influenced her care, one participant (009) said:

See, no, because, uhm, the visits were scheduled ahead and took the necessary precautions—you know, wearing my mask, or I was given a mask when I entered the facility. So, it was not a challenge in the middle of COVID.

##### 2c. Resiliency

Despite citing obstacles with regard to accessing support, navigating online communication, and connecting with their support systems, participants often spoke about moments of resiliency and strength during the postpartum period that affected their breastfeeding experiences. By remaining resilient, participants were able to get through their breastfeeding challenges, using an array of mindfulness techniques (e.g., optimism, awareness, and coping strategies). Being able to focus on their health and the health of their family encouraged the participants to appreciate their postnatal experience in a different way by remaining optimistic. One participant said (006):

Sometimes we plan a lot of stuff, but it didn’t work in that way. So, we have to restart everything and go in another direction. And if the baby is healthy, it’s all okay. If you’re healthy, it’s okay too, right?

By remaining optimistic, many participants were able to cope more successfully with the breastfeeding challenges they were facing. Specifically, one participant (007) felt that her pre-existing support system helped her to feel less isolated throughout quarantine and to cope with the stresses of being pregnant during a precarious time, saying:

I don’t know. . .. Like, it’s been pretty isolated so most of my support has been via the phone. If you have the right support, it’s not as challenging. However, it’s a pandemic. It’s not going to be perfect but, it’s surprisingly—for that much stress, you would assume of being pregnant during a pandemic, it was pretty amazing.

Many participants also demonstrated resilience by becoming aware of and accessing the supports that were still available to them during the COVID-19 pandemic. One participant (003), when talking about herself and her support system remaining positive, had the following to say about its influence on her antenatal experience:

It made my experience much more enjoyable. Like, it made me feel okay and I think that’s everything, actually. I think that I was able to get through my pregnancy the way I was. . ..Like, I got through my pregnancy that way and I kind of had a great pregnancy. And I think that’s because of the support I had with family, friends, and also medical practitioners.

## Discussion

There have been recent commentaries and studies conducted on the potential harmful effects of limited support on breastfeeding practices (Demirci, 2020; Synder & Worlton, 2021; Brown & Shenker 2020). For example, in a commentary by Demirci (2020), it was suggested that lactation support is critical to support a woman in reaching her breastfeeding goals, and that limited access to this support can have damaging effects on parental mental health and wellbeing. This lack of support was further acknowledged by Synder and Worlton (2021), who reported that participants who lacked in-person support from either formal or informal social support were more likely to be at risk of prematurely ceasing breastfeeding during the COVID-19 pandemic. As Vazquez-Vazquez and colleagues (2021) found, participants greatly prefer “face-to-face” support for practical issues regarding their breastfeeding needs, and the results of this study align with this. All of these findings were reiterated by participants interviewed for this current study, who spoke about preferring in-person support as opposed to utilizing online methods of communication and learning. A lack of in-person support was noted as a hinderance to readily receiving credible breastfeeding information and resulted in participants feeling isolated from their support systems.

In this study, one participant had ceased breastfeeding entirely and four participants were no longer breastfeeding exclusively; these participants reported challenges associated with a lack of informal breastfeeding social support and difficulties accessing help with their breastfeeding concerns. As a result, many of the participants in this study struggled with breastfeeding their infant, as they did not have the breastfeeding social support that they felt was necessary to overcome the obstacles they faced—both prior to and during the COVID-19 pandemic—including issues with latching, reassurance they were doing a good job/what is best, and making their own feeding choices. These findings are consistent with other emerging literature. In a study by Brown and Shenker (2020), it was found that 67% of breastfeeding participants in the United Kingdom perceived less social support during the lockdown measures than they had before. Feelings of limited social support were further perpetuated in participants who identified as a minority and/or had experienced IPV. Participants considered to be an at-risk population, including those living in challenging circumstances, those who have experienced IPV, or those who identify as a minority, were more likely to have their breastfeeding practices negatively influenced by COVID-19 lockdown measures. As a result of a perceived lack of breastfeeding social support, many ceased breastfeeding during that time (Brown & Shenker, 2020). Ceulemans and colleagues (2020) found that the COVID-19 pandemic measures caused stress and anxiety for some breastfeeding participants, indicating that personal home contexts also played a large role in breastfeeding practices and outcomes; the findings of this current study reflect this.

Although all participants interviewed for this study cited challenges as a result of the COVID-19 pandemic, some participants reported enhanced perceptions of connectedness. This is similar to findings from other studies. For example, in a study conducted on the general breastfeeding population by Synder and Worlton (2021), the COVID-19 stay-at-home orders had positively influenced some participants by allowing them to be home with their infants without the pressures of working outside of the home. Participants felt that they were able to form deeper and more meaningful connections with their infants and found that their relationship with their partner was more positive (Snyder & Worlton, 2021). Thus, the breastfeeding social support that participants received from their positive relationships allowed them to perceive breastfeeding as a less stressful task. This was also identified by Brown and Shenker (2020), who reported that participants perceived greater support from their partner during the COVID-19 pandemic as their partners were, at times, not able to work and were more present at home, strengthening the relationship between the partner and baby. Some participants in the current study reported similar feelings when their partners were able to be at home and present within the first weeks to months after their infant was born. Some participants identified feeling more supported in their breastfeeding efforts and more positive about their relationship with their partner as a result of this time together. Participants were also able to form meaningful connections with their families and friends using online communication techniques, while some participants cited issues with online communication leading to pressures associated with always being in contact with their families.

Participants demonstrated resiliency through an array of mindfulness strategies by remaining optimistic, becoming aware of the breastfeeding social support that was available, and demonstrating positive coping practices. Through this increased resilience, participants formed stronger bonds with their infants as a result of COVID-19 stay-at-home orders. In addition, remaining positive despite the challenges faced with regard to both breastfeeding and social isolation aided many participants in reaching their breastfeeding goals. Participants worked hard to access both formal and informal breastfeeding social support despite the challenges faced with quarantine measures and service closures.

Brown and Schenker (2020) identified that participants who reported experiencing negative pressure from their families associated with breastfeeding, like frequent visits or judgments about breastfeeding practices, reported feeling less pressure during the COVID-19 pandemic. This decreased in-person contact with certain stressful relationships allowed participants to perceive that they received fewer negative comments around their breastfeeding practices and helped them to feel more confident and competent in their breastfeeding efforts (Brown & Schenker, 2020). This was echoed within the current study, where participants who had strained relationships with family members felt that the limited in-person contact with them aided in their resiliency to meet their breastfeeding goals. Participants also displayed resiliency through their support-seeking efforts, attempting in multiple ways to secure a breastfeeding social support system, especially within the context of a healthcare team. Participants who felt negatively about the lack of in-person support from their families, friends, and healthcare professionals displayed resiliency through managing their expectations about their postnatal experience.

The findings from our study—in the context of the larger body of available evidence—lead to important formal and informal breastfeeding social support recommendations to promote breastfeeding social support for participants from at-risk populations. Formal breastfeeding social support recommendations include: (1) prioritize online options for breastfeeding education classes and ongoing support; (2) prioritize clear communication regarding postpartum care expectations and how to access telephone and/or in-person breastfeeding social support that follows Public Health guidelines; and (3) encourage formal breastfeeding social support to reinforce positive mental health messages (i.e., mindfulness strategies, becoming aware of support available) to allow breastfeeding participants who are from an at-risk population to overcome their breastfeeding challenges. Informal breastfeeding social support recommendations include: (1) encourage women who are at-risk to engage online, or physically distanced, masked and outdoors, with members of their informal support networks (e.g., friends who have previously breastfed) to support breastfeeding; (2) encourage informal breastfeeding support to promote mindfulness techniques (e.g., remaining optimistic) as a solution for participants who are of an at-risk population to overcome their breastfeeding challenges and stressors exacerbated by the pandemic; and (3) prioritize informal breastfeeding social support to reinforce positive messages through encouraging words and breastfeeding preferences (allow participants who are of an at-risk population to make their own breastfeeding choices they are comfortable with).

While heightened public health safety measures and COVID-19 quarantine restrictions offer a means to slow the spread of a deadly virus, the implications for at-risk breastfeeding mothers must be considered. This study has revealed a tension between and the need to balance health and safety and breastfeeding social support among participants who are of an at-risk population. Considering that health and safety issues will be important during recovery from the COVID-19 pandemic and beyond. Future studies should aim to explore: (1) the various ways that participants are of an at-risk population (both personal and systemic oppression); (2) the various influences being of an at-risk population can have on breastfeeding social support; (3) the various resiliency techniques participants of an at-risk population use; (4) consider expanding recruitment strategies to other clinics and/or health services offering emergency supports to ensure a more diverse and representative sample of Canadian participants of a population and; (5) incorporate asking participants about their gender and sex status. This study did not ask participants for additional information regarding what gender and/or sex they referred to themselves as and preferred.

### Limitations

This was a Canadian study; however, it only included participants living in Southwestern, Ontario. The small sample size in conjunction with the fact that we recruited from the clinic increases the likelihood of social desirability response bias. While we utilized honesty demands throughout the interview to try and mitigate this risk, the risk is nonetheless present. In Southwestern, Ontario, between June and September, COVID-19 cases were reported to be decreasing, allowing movement into Phase 2, titled “Restart” of the Ontario COVID-19 Framework. The majority of services were open under this framework, as case totals continued to decrease. Also, interviews were completed over the phone; therefore, the potential to collect observational study data during face-to-face communication might have been missed. This was taken into account during data analysis, as non-verbal cues, emotional triggers, and body language have the potential to provide contextual information to the experiences outlined by the participants. In future studies, researchers should assess additional forms of interviewing that are safe for participants who are of an at-risk population to complete while following public health safety measures (e.g., video chatting platforms or in-person distanced visits). The limited access to services and lack of breastfeeding social support may have also been limited to barriers outside of the COVID-19 pandemic that were not brought forth or assessed during the interviews. Longitudinal data collection where participants are followed up pre-and post-lockdown would reflect more accurately the entirety of the experiences of COVID-19 on postpartum breastfeeding participants of an at-risk population. Lastly, as researchers, we need to understand these results in the context and time in which we are currently living, particularly given that this data was collected during a global pandemic and in the midst of various movements to address systemic oppression (racism, substance use, and gender-based violence). It is important to realize that participants are likely of an at-risk population due to various forms of systemic oppression that are woven into Canadian society, extending beyond the personal experiences of an at-risk population that this study explored.

## Conclusion

The experiences of a small sample size of an at-risk population of postpartum breastfeeding participants in Southwestern, Ontario, during the COVID-19 pandemic has been documented. Our findings provide a foundation for future pandemic-related research to build upon, focusing on the need for breastfeeding social support resources and programs for breastfeeding participants of an at-risk population. Future research should explore how to best respond to participants’ desires for in-person contact during pandemics while balancing public health measures.

## Supplemental Material

sj-docx-1-jhl-10.1177_08903344221091808 – Supplemental material for Experiences of At-Risk Women in Accessing Breastfeeding Social Support During the Covid-19 Pandemic Click here for additional data file. Supplemental material, sj-docx-1-jhl-10.1177_08903344221091808 for Experiences of At-Risk Women in Accessing Breastfeeding Social Support During the Covid-19 Pandemic by Emila Siwik, Samantha Larose, Dalia Peres, Kimberley T. Jackson, Shauna M. Burke and Tara Mantler in Journal of Human Lactation

sj-docx-2-jhl-10.1177_08903344221091808 – Supplemental material for Experiences of At-Risk Women in Accessing Breastfeeding Social Support During the Covid-19 Pandemic Click here for additional data file. Supplemental material, sj-docx-2-jhl-10.1177_08903344221091808 for Experiences of At-Risk Women in Accessing Breastfeeding Social Support During the Covid-19 Pandemic by Emila Siwik, Samantha Larose, Dalia Peres, Kimberley T. Jackson, Shauna M. Burke and Tara Mantler in Journal of Human Lactation

sj-docx-3-jhl-10.1177_08903344221091808 – Supplemental material for Experiences of At-Risk Women in Accessing Breastfeeding Social Support During the Covid-19 Pandemic Click here for additional data file. Supplemental material, sj-docx-3-jhl-10.1177_08903344221091808 for Experiences of At-Risk Women in Accessing Breastfeeding Social Support During the Covid-19 Pandemic by Emila Siwik, Samantha Larose, Dalia Peres, Kimberley T. Jackson, Shauna M. Burke and Tara Mantler in Journal of Human Lactation

sj-docx-4-jhl-10.1177_08903344221091808 – Supplemental material for Experiences of At-Risk Women in Accessing Breastfeeding Social Support During the Covid-19 Pandemic Click here for additional data file. Supplemental material, sj-docx-4-jhl-10.1177_08903344221091808 for Experiences of At-Risk Women in Accessing Breastfeeding Social Support During the Covid-19 Pandemic by Emila Siwik, Samantha Larose, Dalia Peres, Kimberley T. Jackson, Shauna M. Burke and Tara Mantler in Journal of Human Lactation
