# Supplementary material for: Experiences of At-Risk Women in Accessing Breastfeeding Social Support During the Covid-19 Pandemic
Source: J Hum Lact. 2022 Apr 25;38(3):422–32. doi: 10.1177/08903344221091808 (PMC9329748; doi:10.1177/08903344221091808)
Supplement: sj-docx-1-jhl-10.1177_08903344221091808 – Supplemental material for Experiences of At-Risk Women in Accessing Breastfeeding Social Support During the Covid-19 Pandemic [file sj-docx-1-jhl-10.1177_08903344221091808.docx]

Supplemental Material

Semi-Structured Interview Questions.

1. How would you describe your experience in finding a health care team while you were pregnant? Breastfeeding?
2. What obstacles did you face in getting the care you needed?
3. What created those obstacles?
4. What would remove those obstacles?
5. What was helpful to you in terms of accessing health care during your pregnancy? While breastfeeding?
6. Did you feel supported during your pregnancy? Breastfeeding?
7. By your partner?
8. By your family?
9. By your friends?
10. By your health care providers?
11. What contributed to your feelings of support?
12. What made you feel unsupported?
13. How did the level of support you had impact your pregnancy? Breastfeeding?
14. How did the level of support you had impact your experience with health care services?
