# Supplementary material for: Experiences of At-Risk Women in Accessing Breastfeeding Social Support During the Covid-19 Pandemic
Source: J Hum Lact. 2022 Apr 25;38(3):422–32. doi: 10.1177/08903344221091808 (PMC9329748; doi:10.1177/08903344221091808)
Supplement: sj-docx-2-jhl-10.1177_08903344221091808 – Supplemental material for Experiences of At-Risk Women in Accessing Breastfeeding Social Support During the Covid-19 Pandemic [file sj-docx-2-jhl-10.1177_08903344221091808.docx]

Supplemental Material - WHO/UNICEF Breastfeeding Indicators for Mother

Breastfeeding indicators for each child less than 24 months old

What is the date of birth of the child that you breastfed/are breastfeeding?

Day ________________________________________________

Month ________________________________________________

Year ________________________________________________

Since this time yesterday, has your child been breastfed?

Yes

No

*Skip To: WHO04 If Since this time yesterday, has your child been breastfed? = No*

Is breast milk this child's main source of food?

1. Yes
2. No

Since this time yesterday, did your child receive any of the following:

- Vitamins, mineral supplements, medicine
- Plain water
- Sweetened of flavored water
- Fruit juice
- Tea or infusion
- Infant formula
- Tinned, powered or fresh milk
- Solid or semi-solid food
- Oral re-hydration salts (ORS) solution
- Other
- None

*Display This Question:*

*If Since this time yesterday, did your child receive any of the following: = Other*

Please specify:

________________________________________________________________

Since this time yesterday, did your child drink anything from a bottle with a nipple/teat?

1. Yes
2. No

*Display This Question:*

*If Since this time yesterday, did your child drink anything from a bottle with a nipple/teat? = Yes*

Please describe:

________________________________________________________________
