# Supplementary material for: Experiences of At-Risk Women in Accessing Breastfeeding Social Support During the Covid-19 Pandemic
Source: J Hum Lact. 2022 Apr 25;38(3):422–32. doi: 10.1177/08903344221091808 (PMC9329748; doi:10.1177/08903344221091808)
Supplement: sj-docx-3-jhl-10.1177_08903344221091808 – Supplemental material for Experiences of At-Risk Women in Accessing Breastfeeding Social Support During the Covid-19 Pandemic [file sj-docx-3-jhl-10.1177_08903344221091808.docx]

Supplemental Material - Information about Infant

What is your baby's date of birth?

Day ________________________________________________

Month ________________________________________________

Year ________________________________________________

Was your baby born:

Full term (more than 38 week)

Pre term (less than 38 weeks)

What was the gestational age of your baby at birth?

________________________________________________________________

Was your baby admitted to the NICU (Neo-Natal Intensive Care Unit)?

Yes

No

*Display This Question:*

*If Was your baby admitted to the NICU (Neo-Natal Intensive Care Unit)? = Yes*

If you baby was admitted to the NICU, can you please describe when your baby was admitted?

________________________________________________________________

*Display This Question:*

*If Your baby was admitted to the NICU, can you please describe when your baby was admitted? Text Response Is Displayed*

How long was your baby admitted in the NICU (in days)?

________________________________________________________________

What type of delivery did you have?

Uncomplicated vaginal delivery

Vaginal delivery with forceps or vacuum

Planned C-section

Urgent or emergency C-section

What is the gender of your baby?

________________________________________________________________

What was your baby's birth weight in:

Pounds ________________________________________________

Ounces ________________________________________________

Do you breastfeed your baby?

Yes

No

When did you first breastfeed your baby?

________________________________________________________________

Are you currently breastfeeding your baby?

Yes

No

*Display This Question:*

*If Are you currently breastfeeding your baby? = No*

How long did you breastfeed your baby (in weeks)?

________________________________________________________________

Are you breastfeeding your baby exclusively?

Yes

No

*Display This Question:*

*If Are you breastfeeding your baby exclusively? = Yes*

To the above question, if yes how often do you breastfeed?

________________________________________________________________

Do you supplement breastmilk with formula?

Yes

No

*Display This Question:*

*If Do you supplement breastmilk with formula? = Yes*

To the above question, if yes how often?

________________________________________________________________

Is your baby still breastfed?

_________________________________________________________

If yes to the above question, how long has your baby been breastfeeding (in weeks)?

_______________________________________________________________
