# Supplementary material for: Experiences of At-Risk Women in Accessing Breastfeeding Social Support During the Covid-19 Pandemic
Source: J Hum Lact. 2022 Apr 25;38(3):422–32. doi: 10.1177/08903344221091808 (PMC9329748; doi:10.1177/08903344221091808)
Supplement: sj-docx-4-jhl-10.1177_08903344221091808 – Supplemental material for Experiences of At-Risk Women in Accessing Breastfeeding Social Support During the Covid-19 Pandemic [file sj-docx-4-jhl-10.1177_08903344221091808.docx]

Participant Demographic Questionnaire

This questionnaire is intended for women of different backgrounds. What is/are the ethnic or cultural backgrounds(s) you identify with most? (For example: Canadian, English, French, Chinese, East Indian, Italian, German, Scottish, Irish, Cree, Mi'kmaq, Salish, Metis, Inuit, Filipino, Dutch, Ukrainian, Polish, Portuguese, Greek, Korean, Vietnamese, Jamaican, Jewish, Lebanese, Salvadoran, Solami, Colombian, etc.) Please specify as many origins as you like:

________________________________________________________________

 Were you born in Canada?

Yes

No

*Display This Question:*

*If Were you born in Canada? = No*

How long have you lived in Canada? (*please specify years/months,etc.)

________________________________________________________________

 What is your age in years?

________________________________________________________________

 What is your current marital status?

1. Single
2. Married/Common Law/Engaged
3. Divorced/Separated
4. Widowed
5. I prefer not to answer

About how many years have you been with, or were you with, your partner? You may use half years (for example: 2.5 years).

________________________________________________________________

What is the highest level of education that you have completed?

1. Less than high school
2. High school completed
3. Community college and/or journeyman apprenticeship completed
4. University undergraduate degree completed
5. University graduate degree or higher completed
6. I prefer not to answer
7. Other

*Display This Question:*

*If What is the highest level of education that you have completed?  = Other*

 If other, please specify:

________________________________________________________________

What is your current employment status?

1. Employed full-time
2. Employed part-time
3. Unemployed
4. Casual
5. Seasonal
6. I prefer not to answer
7. Other

*Display This Question:*

*If What is your current employment status? = Other*

 If other, please specify:

________________________________________________________________

If unemployed, are you: (please select one)

1. Unemployed but looking for paid work
2. A homemaker (or stay-at-home mother)
3. On maternity or parental leave
4. On sick leave
5. Disabled or unable to work due to health reasons
6. A student
7. I prefer not to answer
8. Other

*Display This Question:*

*If If unemployed, are you: (please select one) = Other*

If other, please specify:

What is your estimated (best guess) yearly combined family income (after taxes are deducted), including employment, government, government cheques, child support, and other sources of income?

1. Less than $19,999
2. $20,000-$49,999
3. $50,000-$99,999
4. Greater than $100,000
5. I prefer not to answer
